# Human eIF3: from ‘blobology’ to biological insight

**DOI:** 10.1098/rstb.2016.0176

**Published:** 2017-03-19

**Authors:** Jamie H. D. Cate

**Affiliations:** 1 Departments of Chemistry and Molecular and Cell Biology, University of California, Berkeley, CA 94720-3220, USA; 2 Lawrence Berkeley National Laboratory, Division of Molecular Biophysics and Integrated Bioimaging, Berkeley, CA 94720, USA

**Keywords:** translation initiation, eIF3, mediator, IRES, eIF4E, eIF3d

## Abstract

Translation in eukaryotes is highly regulated during initiation, a process impacted by numerous readouts of a cell's state. There are many cases in which cellular messenger RNAs likely do not follow the canonical ‘scanning’ mechanism of translation initiation, but the molecular mechanisms underlying these pathways are still being uncovered. Some RNA viruses such as the hepatitis C virus use highly structured RNA elements termed internal ribosome entry sites (IRESs) that commandeer eukaryotic translation initiation, by using specific interactions with the general eukaryotic translation initiation factor eIF3. Here, I present evidence that, in addition to its general role in translation, eIF3 in humans and likely in all multicellular eukaryotes also acts as a translational activator or repressor by binding RNA structures in the 5′-untranslated regions of specific mRNAs, analogous to the role of the mediator complex in transcription. Furthermore, eIF3 in multicellular eukaryotes also harbours a 5′ 7-methylguanosine cap-binding subunit—eIF3d—which replaces the general cap-binding initiation factor eIF4E in the translation of select mRNAs. Based on results from cell biological, biochemical and structural studies of eIF3, it is likely that human translation initiation proceeds through dozens of different molecular pathways, the vast majority of which remain to be explored.

This article is part of the themed issue ‘Perspectives on the ribosome’.

## 1. Introduction

For most genes, the connection between genotype and phenotype requires protein synthesis, translation of the genetic code in messenger RNA (mRNA) into functional proteins. The fundamental mechanisms of how proteins are synthesized on the ribosome are now the subject of sophisticated biochemical and biophysical experiments that build on the insights from the first atomic-resolution structural models of the ribosomal subunits and intact ribosome, including many publications from Venki Ramakrishnan's laboratory [1]. Structural insights into translation are accelerating with the advent of new technologies for cryo-electron microscopy (cryo-EM) [2,3]. However, the myriad ways that cells regulate how and when mRNAs are translated remain to be understood in mechanistic terms.

In eukaryotes, translation initiation serves as a key ‘gatekeeper’ to protein production levels. A canonical model for translation initiation has emerged that requires over a dozen translation initiation factors or eIFs [4]. Cellular mRNAs are capped with a 7-methylguanosine (m^7^G) structure at the 5′ end recognized by translation initiation factor eIF4E. Once assembled with eIF4E in the context of a larger eIF4F complex, mRNAs are recruited to the small (40S) subunit of the ribosome that harbours additional initiation factors in a 43S complex. Finally, helicases in eIF4F help the 48S pre-initiation complex scan the mRNA from the 5′ end to the appropriate start codon, usually but not always the first encountered [5]. The two initiation factors eIF4E and eIF2 are known to serve as major focal points of translation regulation. Multiple pathways that sense cellular metabolism and stress converge on eIF4E to determine the level of translation from cellular mRNAs, and include the mechanistic target of rapamycin complex 1 and MAPK-interacting kinases Mnk1 and Mnk2 [6,7]. In parallel and not entirely independently, the nutritional state of the cell and stress control the levels of active eIF2, which delivers initiator tRNA to translation pre-initiation complexes [5,6,8]. These pathways, when perturbed, can contribute to human diseases including cancer [7,9,10].

Although eIF4E acts at the start of the scanning mechanism of translation initiation, many variations have been identified that tune the levels of protein synthesis for select mRNAs, including upstream open reading frames (uORFs) that modulate the expression of a downstream ORF [11]. In the case of yeast *GCN4* and human *ATF4*, uORFs connect levels of translation of these critical transcription factors directly to the nutritional state of the cell [6,11]. Another prominent example common among positive-strand RNA viruses is the use of structured RNA elements in the 5′ untranslated regions (5′UTRs) preceding virally encoded ORFs. These structured regions—termed internal ribosome entry sites or IRESs—recruit the translational machinery directly to the start codon [4]. Viral IRES elements are thought to help viruses circumvent immune surveillance mechanisms that repress general translation, mediated by reducing active levels of eIF2, the initiation factor that loads initiator tRNA^Met^_i_ at the start codon in the 48S complex [12–14]. Other sequence- and structure-specific mechanisms for regulating translational efficiency of classes of mRNAs are beginning to emerge, including, for example, 5′-terminal oligopyrimidine sequences in mRNAs [15], TISU elements [16], structured RNA elements in histone mRNAs [17] and in the 5′UTRs of homoeobox (Hox) genes [18], RNA elements that lead to ribosome shunting [16] and other translational enhancers [19]. Finally, gathering evidence indicates that mRNAs can be post-transcriptionally modified with pseudouridine, *N*^6^-methyladenosine and *N*^1^-methyladenosine [20]. How these regulate translation initiation remains unclear. However, evidence suggests that *N*^6^-methyladenosine can stimulate translation initiation in conditions in which eIF4E is limiting [20].

Here I describe how recent advances in understanding the structure and function of translation initiation factor eIF3 help illuminate the wide variety of initiation mechanisms that are likely to exist in eukaryotes. This review focuses primarily on mammalian eIF3, with reference to other organisms such as the yeast *Saccharomyces cerevisiae* to highlight universal features of eIF3 structure and function. In most multicellular eukaryotes, eIF3 is composed of 13 subunits, named eIF3a–eIF3m [21–23]. Land plants, which diverged from animals about 1.6 billion years ago [24], retain a 13-subunit eIF3 complex [25], and *Arabidopsis* and wheat germ extracts have been used extensively to study translation initiation [25]. Filamentous fungi—less evolutionarily diverged from metazoans—also contain a 13-subunit eIF3 [23]. Although *S. cerevisiae* eIF3 has contributed greatly to our understanding of eIF3 function [5], it is composed of only six subunits [21]. These six subunits (eIF3a, eIF3b, eIF3c, eIF3g, eIF3i and eIF3j) are conserved with those in humans, but may have evolved distinct regulatory roles in yeast when compared with multicellular eukaryotes, for example in interactions with other initiation factors [26,27].

## 2. Discovery of eIF3 and its roles in general translation initiation

Mammalian eIF3, originally isolated by biochemical fractionation and functional reconstitution of translation initiation, is the largest of the translation initiation factors, with a molecular mass of approximately 800 kDa. Most of its subunits were identified by the mid-1970s. The Staehelin and Blobel laboratories were the first to note the multiple protein composition of eIF3 [28,29], and identified nine to 10 separate polypeptides [29,30]. Hershey was able to identify 11 bands in eIF3 purified from rabbit reticulocytes [31]. Its full composition, however, took many decades to decipher owing to the presence of one labile subunit—eIF3j [32]—and a second with anomalous mobility on denaturing gels—eIF3m [22,33]—that may also be labile in the eIF3 complex [34,35].

Although the composition of mammalian eIF3 took significant effort to establish, its central roles in translation initiation were identified in the 1970s using *in vitro* reconstitution of translation on natural mRNAs and viral genomes with biochemically fractionated factors. The Anderson laboratory initially described eIF3 (IF-M_3_) as required for translation of natural mRNAs but not poly-U [36]. A similar activity was described by Heywood [37], and was additionally shown to be required for ribosome recycling [38]. In parallel efforts, the Staehelin laboratory showed that eIF3 (IF-E_3_) is required for binding Met-tRNA^Met^_i_ to the 40S subunit in the absence of mRNA [28], the first evidence for the role of eIF3 in the formation of the 43S pre-initiation complex identified by Hunt & Jackson [39] and Hirsch [40,41]. Making the first connection between eIF3 and viral translation, Strycharz *et al.* [42] showed that eIF3 is essential for translation of the encephalomyocarditis viral RNA.

**Figure 1.**
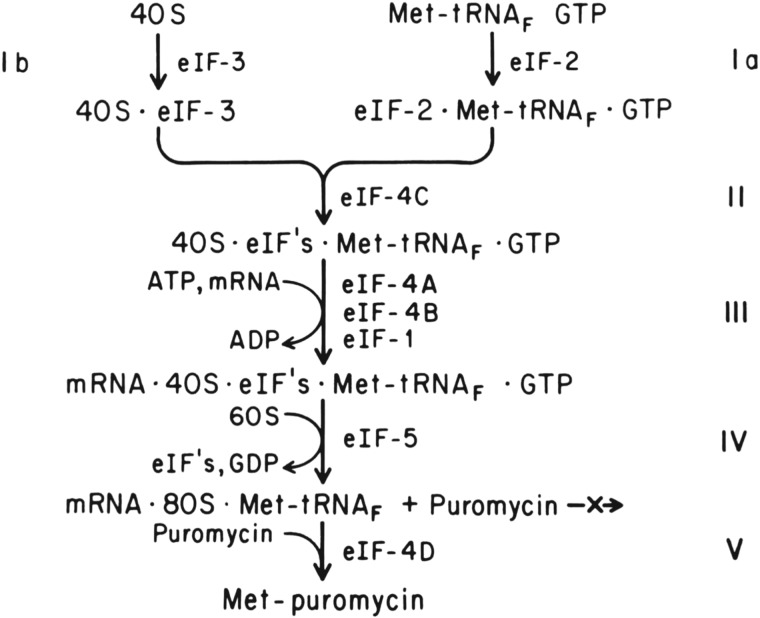
Early model for the mechanism of translation initiation. The nomenclature of initiation factors changed as follows [52]: eIF-1 now eIF1, eIF-2 now eIF2, eIF-3 now eIF3, eIF-4A now eIF4A, eIF-4B now eIF4B, eIF-4C now eIF1A, eIF4-D now eIF5A, eIF-5 now eIF5. Figure from [44].

During subsequent years, multiple groups refined the purification of most of the translation initiation factors, allowing for a much deeper analysis of initiation mechanisms [43–49]. Of particular note with respect to eIF3 function, purified eIF3 could be phosphorylated [50] or reductively methylated [44,51] *in vitro* and tracked through the various steps of initiation. Using radiolabelled eIF3, these groups showed eIF3 binds the 40S subunit in the absence of other translation initiation factors, stabilizes eIF2/Met-tRNA^Met^_i_/GTP binding to the 40S subunit, is required for maximal binding of natural mRNAs to the 40S subunit, inhibits 60S subunit joining and is released upon 80S initiation complex formation [44,50,51] (figure 1). Thus, even at this early stage in understanding eukaryotic translation initiation—before the role of the eIF4F complex had been established [46–49] and the remaining core initiation factor, eIF5B, had been found [53]—the central importance of eIF3 to translation initiation was apparent.

## 3. Early insights into eIF3 structure and binding to pre-initiation complexes

**Figure 2.**
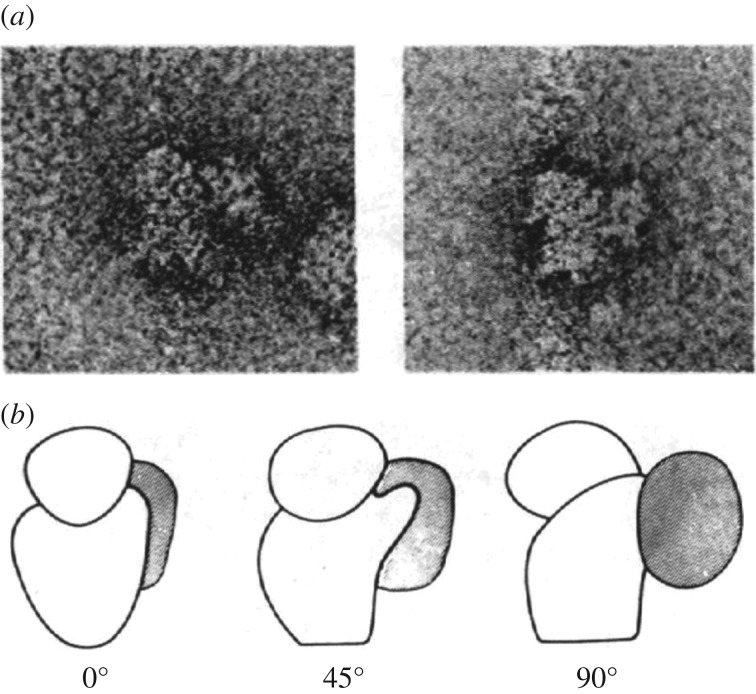
Negative stain EM images of ‘native’ 40S subunits. (*a*) Examples of negatively stained particles. (*b*) Model of eIF3 bound to the 40S subunit, in three orientations. These 40S subunit preparations retained eIF3 bound to the platform region. From [54].

Structurally, James Lake and collaborators showed quite soon after its discovery that eIF3 associates with the platform of the 40S ribosomal subunit [54] (figure 2), but its overall architecture remained unclear. Bielka and co-workers first proposed eIF3 to be flat and triangular in shape [55,56]. The first three-dimensional reconstruction of eIF3 bound to the 40S subunit determined by Joachim Frank's laboratory, at approximately 5 nm resolution, more clearly revealed eIF3 projecting away from the platform and the interface between the two ribosomal subunits [57]. From these early structural results, it was not yet clear how eIF3 accomplished its roles in translation initiation or recycling, although Bielke's group found it to be located in close proximity to eIF2, which bound between the head and body of the 40S subunit [58]. In 2005, the Doudna and Nogales groups published the first cryo-EM reconstruction of eIF3, revealing its five-lobe architecture for the first time [59]. This reconstruction at approximately 30 Å resolution coupled with additional EM images identified the possible location of eIF4G binding to eIF3, and the position of the HCV IRES bound to eIF3 off the ribosome. Notably, the authors combined these reconstructions with modelling based on the three-dimensional reconstruction of negative-stain images of eIF3 bound to the 40S subunit [57] to provide a framework for understanding eIF3 structure and function. Although state-of-the-art at the time, the low resolution led the authors to contour the EM map to enclose a volume consistent with the molecular mass of the imaged particle, in this case approximately 800 kDa in mass. However, the choice of contour level is directly impacted by the assumption that a particle is rigid at the resolution of the reconstruction. As described in §4, eIF3 turns out to be highly flexible outside of its core, a feature of the complex not appreciated structurally for many years.

With the identification of the genes for nearly all of the subunits in eIF3 [60], it was possible to identify its evolutionary similarity to the COP9 signalosome—or CSN complex—and 26S proteasome regulatory lid complex [61–65]. However, advances into the mechanisms of eIF3 function during these years occurred primarily by using genetics in *S. cerevisiae* and biochemistry to study the conserved subunits eIF3a, b, c, g, i and j. Hinnebusch and co-workers used yeast to map interactions between eIF3 domains [66–68], and to identify a previously unknown ‘multifactor complex’ (MFC) between eIF3, eIF1, eIF2, eIF5 and initiator tRNA(Met) as a *bona fide* translational intermediate in the assembly of translation initiation complexes *in vivo* [67–69]. Several following papers identified contacts between eIF3 subunits, other translation initiation factors in the MFC, eIF4G and the 40S subunit important for translation initiation in yeast [70]. However, while many of these interactions may be conserved across eukaryotes, some aspects of translation initiation have likely diverged. In addition to the larger number of subunits in eIF3, the MFC in humans seems to involve different interactions between the eIFs than in yeast, and furthermore may participate in multiple pathways of delivering Met-tRNA_i_ to the 40S subunit during 43S complex assembly [71].

## 4. Insights into mechanisms of mammalian eIF3 in translation initiation

Attempts to biochemically dissect mammalian eIF3 lagged those in yeast, owing to the difficulty in reconstituting the complex to enable mutagenesis. However, purified factors enabled a number of new insights into eIF3 function. Studies with purified translation factors helped identify the biochemical role of eIF3 in preventing 40S and 60S subunit association after recycling, an activity that required the ternary complex of eIF2/GTP/Met-tRNA_i_ [72]. Purified translation factors also enabled the first mapping of translation factor requirements for viral IRES translation initiation, in particular how eIF3 and other factors interact with different types of IRES [73,74]. To begin dissecting mammalian eIF3, the Hershey and Sonenberg groups used baculovirus expression in insect cells to reconstitute mammalian eIF3 subassemblies comparable to the yeast eIF3 complex [75] and eIF3 with up to 11 subunits in composition [34]. Using a complex of six subunits—interestingly lacking two universally conserved in eukaryotes, eIF3g and eIF3i—the Sonenberg group could demonstrate that this minimal mammalian eIF3 (eIF3a, b, c, e, f and h) assembled translation initiation complexes *in vitro* [34]. Unfortunately, it has not been possible to test the function of this six-subunit complex in multicellular eukaryotes, as several of the non-essential subunits *in vitro* are essential *in vivo* [76–78].

**Figure 3.**
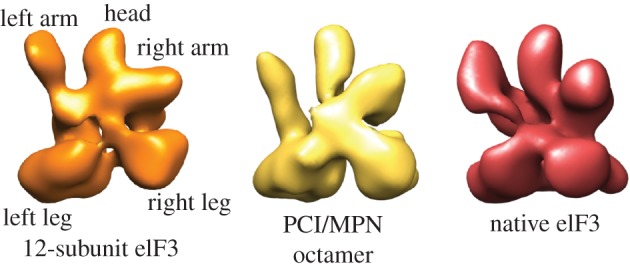
Structural core of human eIF3. Comparisons of negatively stained reconstructions of recombinant 12-subunit eIF3 (approx. 700 kDa), eight-subunit core of eIF3 (approx. 400 kDa), and the cryo-EM reconstruction of natively purified intact eIF3 (approx. 800 kDa). From [81].

My laboratory began working on determining the structure of human eIF3 in the late 2000s, making the choice to reconstitute human eIF3 using *Escherichia coli* as an expression host rather than using baculovirus expression or native purification. Although this precluded isolating eIF3 with possible post-translational modifications [33,79], it would allow us to functionally dissect eIF3 with far more precision than we could with natively purified eIF3 [80], and potentially with high enough yields for structural biology. We found that it was possible to functionally reconstitute human eIF3 in *E. coli*, minus two flexible regions—the C-terminus of eIF3a and N-terminus of eIF3c—and the first four amino acids of eIF3a [81]. This reconstitution revealed the eight-subunit structural core of eIF3, related in architecture to the proteasomal lid and COP9 signalosome through its PCI/MPN (proteasome, CSN, eIF3/Mpn1 Pad1-N-terminal) domain containing subunits: eIF3a, eIF3c, eIF3e, eIF3f, eIF3h, eIF3k, eIF3l and eIF3m [65]. Remarkably, the core of eIF3, about 400 kDa in mass, had the same overall shape as intact eIF3 (figure 3). Thus, from these first reconstitutions, it became abundantly clear that at least half of eIF3 was conformationally dynamic [81] and would require imaging in intact pre-initiation complexes to determine its structure to high resolution. Notably, hints of eIF3 flexibility had already been observed with respect to eIF3j, which approaches the mRNA decoding centre of the 40S subunit from the side facing the 60S subunit, in nearly the opposite direction to the known binding site of eIF3 to the 40S subunit platform [82]. Our reconstitutions of eIF3 using *E. coli* also revealed that eIF3 function for at least HCV IRES-mediated translation initiation would require all of the 12 stably associated subunits of eIF3, minus possibly only eIF3j; subassemblies lacking eIF3b, eIF3g and eIF3i were not functional [81].

**Figure 4.**
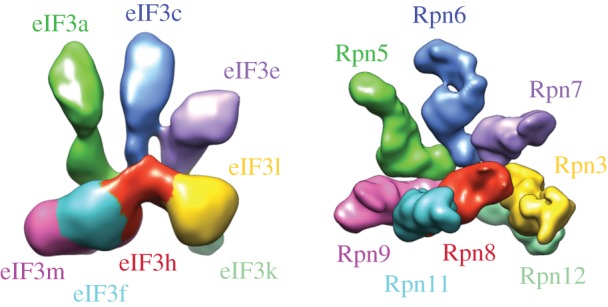
Cryo-EM reconstruction of the 8-subunit core of human eIF3. The position of the eight subunits in the core of eIF3, left, were determined by N-terminal tagging and comparison with the proteasomal lid, right. From [27].

Using *E. coli* reconstituted eIF3 also allowed us to map the position of subunits in the mammalian complex for the first time [27]. We used N-terminal MBP or GST tags and comparisons with the proteasome lid architecture [83–85] to identify the locations of each of the eight core subunits, along with the approximate positions of eIF3d and eIF3j N-termini (figure 4). We also identified binding between the eIF3 core and eIF1, interactions possibly conserved in the MFC [70,71]. Interestingly, mammalian eIF3 also interacts with eIF1A, perhaps through interactions that differ between yeast and humans. In yeast, contacts between eIF1A and yeast eIF3 occur in flexible and less-conserved N-terminal regions of eIF3c deleted in the reconstituted human eIF3 complex [26,27].

## 5. Towards atomic-resolution structural understanding of eIF3 function

**Figure 5.**
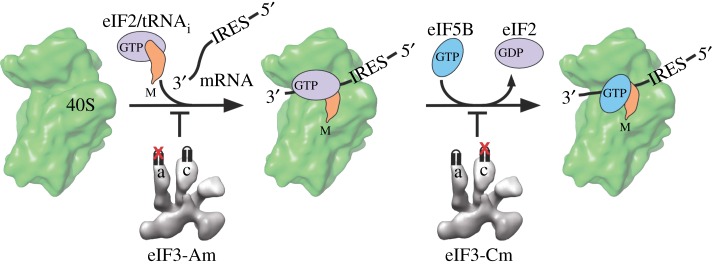
Roles of helix–loop–helix RNA-binding motifs in eIF3 in HCV IRES-mediated translation. The motif in subunit eIF3a mediates start codon recognition, whereas the motif in eIF3c contributes to a later step involving eIF5B. From [27].

The cryo-EM reconstruction of the human eIF3 core complex was determined at quite low resolution, of about 15–20 Å (figure 4), but did allow homology modelling of the PCI/MPN subunits and nearby alpha-helical repeat segments, by comparison with atomic-resolution structures of subunits within the proteasome lid [27,86]. Using homology models within the low-resolution cryo-EM map allowed us to use a resolution at the level of ‘blobology’ to identify two previously unrecognized RNA-binding motifs in eIF3 [87]. These two predicted RNA-binding helix–loop–helix (HLH) motifs are located at the N-terminus of eIF3a and near the N-terminus of eIF3c, just C-terminal of the less-conserved region of eIF3c beginning near amino acid 302. We could show by mutagenesis that the motif in subunit eIF3a is critical for eIF3 binding to both the HCV IRES, and to the 40S ribosomal subunit, and seems to control initial recognition of the AUG start codon. By contrast, the RNA-binding motif in eIF3c is less critical for 40S subunit and IRES binding, but influences the later step of eIF5B-mediated 60S subunit joining [87] (figure 5).

**Figure 6.**
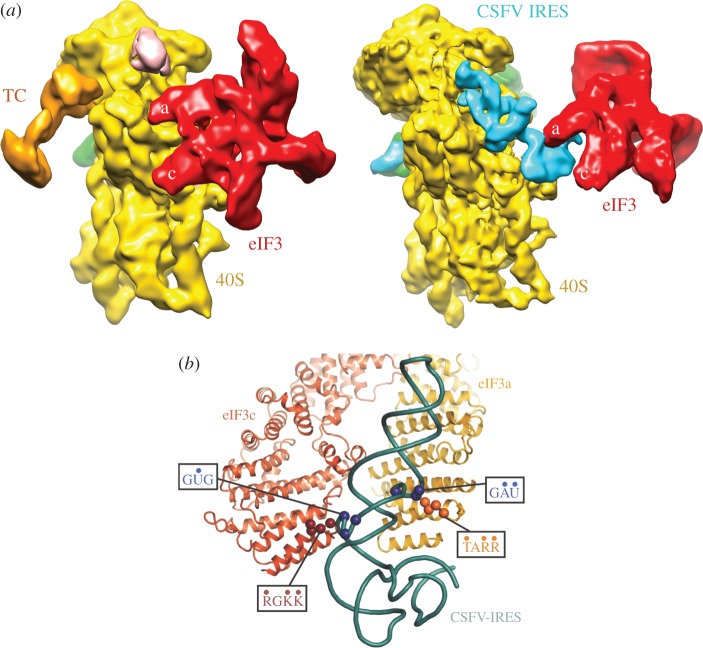
Different modes of eIF3 binding to pre-initiation complexes. (*a*) Left: a 43S pre-initiation complex. Right: eIF3 bound to a classical swine fever virus (CSFV) IRES-40S complex. Figures adapted from [88,89]. (*b*) Model of the CSFV IRES interactions with subunits eIF3a and eIF3c. From [90].

Given the size of the RNA-binding motifs, it was puzzling that the HLH motif in eIF3a is important both for binding to the HCV IRES and to the 40S ribosomal subunit. How could a small protein motif bind both the HCV IRES and the 40S subunit at the same time? Sterically, this seemed implausible, and we wondered if it might be involved in serial interactions during pre-initiation complex formation. Remarkably, two cryo-EM reconstructions of mammalian eIF3 bound to the 40S subunit, one in a 43S-like complex [88] and a second in complex with the HCV-like CSFV IRES and 40S subunit [89], revealed two different modes of eIF3 binding. In the 43S complex, eIF3 binds the platform of the 40S subunit, whereas the IRES displaces eIF3 from the 40S through direct IRES RNA–protein interactions (figure 6*a*). These results helped explain how the HLH motif in eIF3a could be responsible both for binding to the 40S subunit and HCV IRES. In a subsequent structural investigation of eIF3 bound to the 40S subunit that combined the approximately 12 Å cryo-EM reconstruction of the 43S complex [88], X-ray crystal structures of yeast eIF3 subunits and subcomplexes and cross-linking–mass spectrometry, it was possible to model the interaction between the HCV IRES and subunits eIF3a and eIF3c at the amino acid and RNA nucleotide resolution (figure 6*b*) [90].

**Figure 7.**
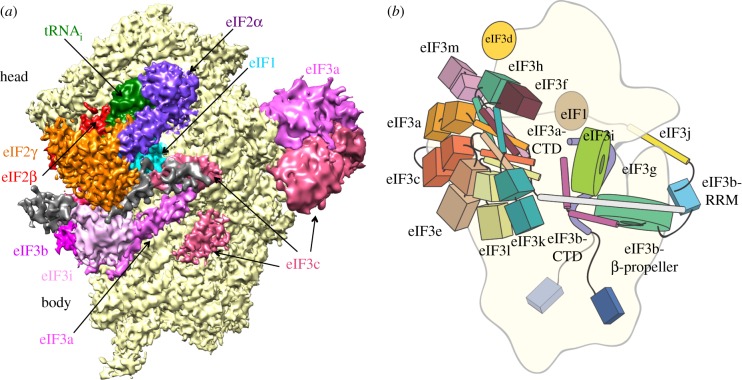
Models of eIF3 wrapping around the entirety of the 40S ribosomal subunit. (*a*) Yeast 48S pre-initiation complex, viewed from the perspective of the 60S subunit interface. Adapted from [93]. (*b*) Model of mammalian eIF3 bound to the 40S subunit, viewed from the solvent side of the 40S subunit. Updated from [90].

Structural insights into eIF3 and translation initiation are continuing at a rapid pace, using both yeast and mammalian systems for cryo-EM reconstructions [91–93]. One of the most striking findings of these cryo-EM reconstructions is the fact that eIF3 effectively wraps around the 40S subunit, reaching from under the mRNA decoding site—the binding location for eIF1A [93] (figure 7*a*)—to the 40S subunit platform [91–93] and then entirely around the solvent side of the 40S subunit reaching past the mRNA entry tunnel [90–93] (figure 7*b*). The core of mammalian eIF3 is the most distal part of the eIF3 complex from the 80S ribosomal subunit interface (figure 6*a*), with extensions from the core extending to contact multiple regions of the pre-initiation complex (figure 7). Furthermore, these extensions are highly dynamic even in the context of pre-initiation complexes, both in yeast and mammals [93,94]. These cryo-EM reconstructions will enable many new biochemical and genetic explorations of eIF3 function in translation in the years to come.

## 6. Roles of eIF3 as the ‘mediator’ of translation initiation

The role of eIF3 in stimulating translation initiation from structured viral IRES elements [4] by binding directly to the highly structured HCV IRES and not the 40S ribosomal subunit [89] raises questions of how general this mechanism of initiation may be. To test whether eIF3 controls alternative modes of translation initiation on cellular transcripts, we used photoactivatable-ribonucleoside-enhanced cross-linking and immunoprecipitation [95] with 4-thiouridine to identify transcripts that specifically interact with eIF3 in human 293T cells. Although we were concerned that we may only purify eIF3 cross-linked near the start codon [96], surprisingly, we found highly discrete sites of cross-linking to approximately 500 cellular transcripts [97]. Of these, most sites of cross-linking occurred at the 5′UTR as would be expected for the role of eIF3 in initiation. These mRNAs fell into distinct regulatory categories related to cell proliferation, the cell cycle, apoptosis and differentiation. Remarkably, we found that eIF3 binding to secondary structural elements in the 5′UTR of select mRNAs could not only activate translation, but could also repress translation. For example, translation of *JUN* mRNA, encoding the immediate early response transcriptional regulator c-Jun in the AP-1 transcription factor, is activated by eIF3. By contrast, *BTG1* mRNA, encoding a transcriptional regulator B-cell translocation gene 1 that promotes terminal differentiation, is repressed by eIF3. Both these activities of eIF3 depend on the 5′ 7-methylguanosine cap on the mRNA [97].

The eIF3-binding sites within the mRNAs that cross-linked to eIF3 interact with four of its subunits—eIF3a, eIF3b, eIF3d and eIF3g—in all possible combinations of 1,2, 3 and 4 of the subunits. Although it is possible that the differences in cross-linking pattern could be due in part to the distribution of uridines in the sequences (i.e. the sites of 4-thiouridine incorporation), the pattern of cross-linking suggests that there may in fact be dozens of different ways that mRNAs are recruited to the 40S subunit for translation initiation, hints of which have already been revealed in cryo-EM reconstructions of the 43S pre-initiation complex and classical swine fever virus (CSFV) IRES pre-initiation complex (figure 6) [88,89]. In support of this idea, two of the mRNAs we characterized in depth—*JUN* and *BTG1*—harbour distinct secondary structures in their 5′UTRs that either activate or repress translation, respectively [97].

## 7. eIF3 possesses a 5′ m^7^G cap-binding subunit

**Figure 8.**
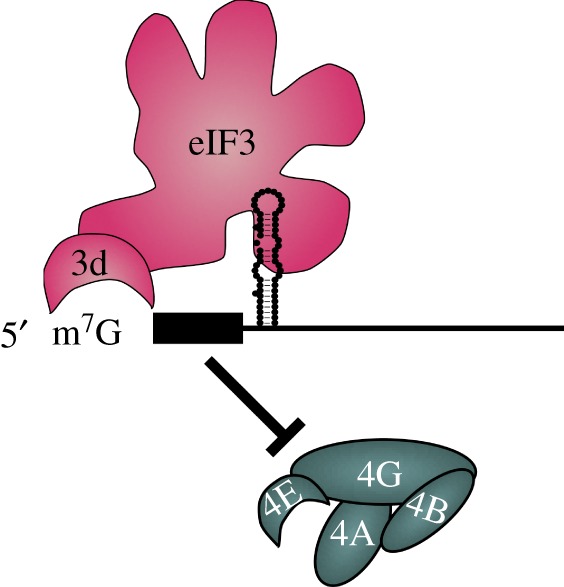
Mechanism of 5′ m^7^G cap-binding by eIF3 in translation of *JUN* mRNA. Upon binding the specific stem–loop in the *JUN* 5′UTR, eIF3d binds the 5′ cap to promote translation. An RNA element near the 5′ cap prevents eIF4F activation of *JUN* mRNA translation. Updated from [102].

The 5′ cap dependence of *JUN* mRNA translation activation requires a mechanistic explanation. Why, when the eIF3-binding secondary structure in the *JUN* mRNA is mutated thereby disrupting translation of c-Jun, does the eIF4F cap-binding complex not substitute to drive translation initiation? More generally, conditions of stress or nutrient deprivation lead to eIF4E sequestration and inactivation, yet many transcripts in the cell continue to be translated well [98], including *JUN* [99–101]. By analysing *JUN* mRNA translation initiation, we discovered that the eIF4F complex is not involved in *JUN* translation, which instead depends on a previously unknown 5′ cap-binding activity in subunit eIF3d [102]. Using the same cross-linking approach originally pioneered by Nahum Sonenberg to identify eIF4E [46,103], we found that eIF3d binds specifically to the 5′ m^7^G cap. However, cap-binding by eIF3d does not seem to occur for mRNAs in general. We determined the X-ray crystal structure of the eIF3d cap-binding domain and found a striking homology to the recently identified DXO family of cap exonucleases [104]. However, eIF3d has an ‘RNA gate’ motif that blocks access to the 5′ cap-binding pocket. Only upon allosteric activation by specific RNA structures—exemplified by the eIF3-binding site in the *JUN* 5′UTR—does eIF3d bind the cap to promote translation [102]. Notably, the *JUN* mRNA also includes an RNA element near the 5′ cap that prevents eIF4F binding and activation of translation, likely to enforce the eIF3 dependence of *JUN* translation to specific biological contexts (figure 8). Inhibition of eIF4F activity to promote alternative translation pathways is also required for the translation of homoeobox mRNAs [18]. The cap-binding domain in eIF3d—universally conserved in multicellular eukaryotes—likely serves to drive the translation of select regulatory mRNAs, many of which may also encode RNA structures that prevent the action of eIF4F in translation initiation [102].

## 8. Conclusion

The surprising discovery that eIF3 can activate or repress translation of specific mRNAs bears a striking parallel to the role of the mediator complex in transcription [97,105]. In the future, it will be important to identify *trans*-acting factors that are likely required for the function of eIF3 in transcript-specific translation regulation. Further efforts to understand how eIF3 stimulates translation of mRNAs marked with *N*^6^-methyladenosine [106] will also require new mechanistic insights. Finally, all of these newly identified biological roles for eIF3 will require new structural insights into both the canonical scanning model, and into the molecular events underlying the myriad additional translational regulatory pathways that are continuing to be uncovered.
